# Miscellaneous Items

**Published:** 1845-11-01

**Authors:** 


*Miscellaneous Items.*—Dr. Jarvis the inventor of the "Surgical Adjuster" is said to have been successful in his efforts to bring his invention into favorable notice in Great Britain. The English will, perhaps, some time be compelled to recognise the supremacy of Yankee ingenuity even in matters of Science.

Dr. Barbour of St. Louis, professor in the Kemper College, in the last No. of the St. Louis Journal, sustains the plan of treating fevers with opium in doses of four or five grains, advocated in a previous No. of that Journal by Dr. Henry, noticed by us in our last number.

Dr. Lawson, Editor of the Western Lancet, now abroad on a foreign tour, is writing a series of very interesting letters for his Journal, which we regret we cannot, for lack of space, transfer to our columns.

The St. Louis Journal expresses itself warmly in favor of a National Convention, and advocates the removal of the power to confer Degrees from Collegiate Institutions, and its concentration in a National Board. The Western Lancet, also urges the importance of the Convention; and, from a quotation in the latter, we perceive that the Medical Examiner does so also.

The new Edifice for the Medical College at Cleveland, is to be in readiness for the course of lectures now at hand. The pictorial representation accompanying the circular recently issued, presents an imposing appearance.

